# Population Diversification in *Staphylococcus aureus* Biofilms May Promote Dissemination and Persistence

**DOI:** 10.1371/journal.pone.0062513

**Published:** 2013-04-30

**Authors:** Victoria J. Savage, Ian Chopra, Alex J. O’Neill

**Affiliations:** Antimicrobial Research Centre, School of Molecular and Cellular Biology, University of Leeds, Leeds, United Kingdom; Université d’Auvergne Clermont 1, France

## Abstract

The biofilm mode of growth can lead to diversification of the bacterial population by promoting the emergence of variants. Here we report the identification and characterization of two major subpopulations of morphological variants arising in biofilms of *S. aureus*. One of these lacked pigmentation (termed white variants; WVs), whilst the other formed colonies on agar that were larger and paler than the parental strain (termed large pale variants; LPVs). WVs were unable to form biofilms, and exhibited increased proteolysis and haemolysis; all phenotypes attributable to loss-of-function mutations identified in the gene encoding the alternative sigma factor, *sigB.* For LPVs, no differences in biofilm forming capacity or proteolysis were observed compared with the parental strain. Genetic analysis of LPVs revealed that they had undergone mutation in the accessory gene regulator system (*agrA*), and deficiency in *agr* was confirmed by demonstrating loss of both colony spreading and haemolytic activity. The observation that *S. aureus* biofilms elaborate large subpopulations of *sigB* and *agr* mutants, both genotypes that have independently been shown to be of importance in staphylococcal disease, has implications for our understanding of staphylococcal infections involving a biofilm component.

## Introduction

A growing body of evidence supports the idea that even monospecies biofilms comprise phenotypically and genotypically heterogeneous populations [1], [2], [3], [4]. This heterogeneity suggests that the biofilm mode of growth prompts diversification of the bacterial population by promoting the emergence of variants, and indeed several bacterial species (e.g. *Pseudomonas aeruginosa*, *Streptococcus pneumoniae* and *Staphylococcus aureus*) have been shown to elaborate morphological variants during biofilm growth [1], [5], [6], [7]. This process is attributable, at least in part, to the increased genetic plasticity of bacterial biofilm populations, which exhibit enhanced mutability and recombination relative to their planktonic counterparts [3], [8], [9].

A subset of the variants arising during biofilm growth of bacterial pathogens may possess characteristics with relevance to our understanding and management of bacterial disease. For example, we and others have shown that antibiotic-resistant mutants arise with greater frequency in bacterial biofilms [3], [8], [9]. It has also been demonstrated that the biofilms of some species contain large subpopulations of small colony variants (SCVs) [10], [11]; such variants exhibit reduced susceptibility to a variety of antimicrobial agents [6], [12], and may play a key role in establishing chronic infection [13].

Here we investigated the emergence of variants during biofilm growth of the important human pathogen *S. aureus*, focussing on detection of morphological variants that could be visually distinguished from the parental strain. We report the identification of two types of morphological variant that represent major subpopulations of both static and continuous-flow biofilms. Based on their phenotypes, we propose that these subpopulations play important and distinct roles during staphylococcal infection.

## Materials and Methods

### Bacterial Strains and Reagents

Laboratory strains *Staphylococcus aureus* SH1000 [14], [15], RN4220 (*agr*-deficient control) [16], and the morphological variants recovered in this study (WV1, WV2, LPV1, LPV2) were maintained on Mueller-Hinton agar (MHA) and routinely cultured in Mueller-Hinton broth (MHB; both from Oxoid, Basingstoke, UK). All chemicals and reagents were from Sigma-Aldrich (Dorset, UK).

### Identification of Morphological Variants Arising in Biofilm Cultures

Two biofilm models were used in this study. The cellulose disk static (CDS) biofilm model was as described [17]. Briefly, mixed ester cellulose disks (25 mm diameter, 0.22 µm pore size; Millipore, Billerica, USA) soaked in human plasma (4% v/v [Sera Laboratories International, Bolney, UK] in 0.05 M carbonate bicarbonate buffer [pH 9.6]) were utilised as the substratum for biofilm formation by incubating inoculated disks on brain heart infusion agar (Oxoid) for 48, 72, 96 or 144 hrs. The Sorbarod biofilm model [8] is a constant flow system in which biofilms form under shear forces. Cylindrical filters composed of compacted cellulose fibres (Sorbarod filters, with a diameter of 10 mm and length of 20 mm [Ilacon, Kent, UK]), coated in human plasma as above, were utilised as the biofilm substratum; in this case biofilms were grown for 96 hrs. To determine the proportion of morphological variants in the nonadherent and adherent phase of the biofilms, bacteria liberated during a saline wash and following cellulase treatment [18], respectively, were enumerated on MHA after 24 hrs of growth at 37°C.

### Phenotypic Characterization of Morphological Variants

To examine the proteolytic properties of the morphological variants, aliquots (10 µl) of overnight (16 h) cultures were spotted onto milk agar (2% [w/v] pasteurised milk) to visualise casein proteolysis [19]. Plates were incubated at 37°C overnight, and were then flooded with 1% (v/v) HCl to precipitate undigested casein. Haemolysis was examined by spotting aliquots (10 µl) of overnight cultures onto fresh blood agar; α-haemolysin activity was visualised after incubation overnight at 37°C, and plates were then placed at 4°C for 16 hrs to visualise β-haemolysin activity [20]. Evaluation of colony spreading, a marker of biosurfactant/phenol soluble modulin (PSM) production, was performed as described by Tsompanidou *et al.* [21].

### Genetic Characterization of Morphological Variants

Genomic DNA was extracted using the PurElute Bacterial Genomic kit from EdgeBiosystems (Maryland, US), with some modification to the manufacturer’s protocol. Bacteria were harvested from volumes (2 ml) of 16 h cultures, washed in TE buffer, resuspended in Spheroplast buffer supplemented with lysostaphin (100 µg/ml), and incubated at 37°C for 1 hr with gentle mixing every 15 min. Samples were then processed as per the manufacturer’s instructions, with the addition of proteinase K (100 µg/ml) to the Extraction buffer.

The genetic basis of the WV phenotype was investigated by PCR amplification and DNA sequencing of the *sigB* and *rsbU* genes. The genotype of LPVs was determined by whole genome sequencing performed by BGI Genomics (Hong Kong) using Illumina technology. Short-read alignment to the *S. aureus* SH1000 reference sequence and identification of putative mutations was performed by BGI Genomics using SOAPaligner [22]. PCR amplification and DNA sequencing were used to confirm the presence of putative mutations.

## Results

**Figure 1:**
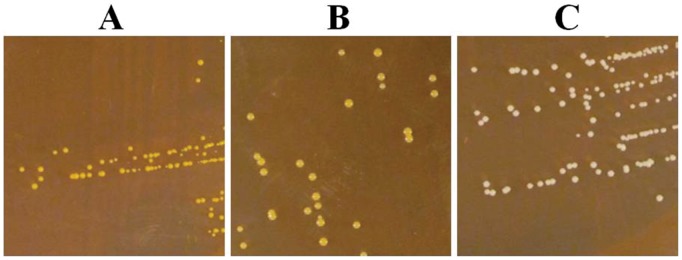
Colony morphology of *S. aureus* SH1000 (parental strain) (A), LPVs (B) and WVs (C).

Colonies exhibiting morphological variation were initially detected upon plating onto agar dilutions of *S. aureus* SH1000 biofilm cultures grown using the cellulose disk static (CDS) biofilm model. Two colony morphologies distinct from the parental strain were identified; nonpigmented colonies, which we designated white variants (WVs), and large pale variants (LPVs) (Figure 1).

**Figure 2:**
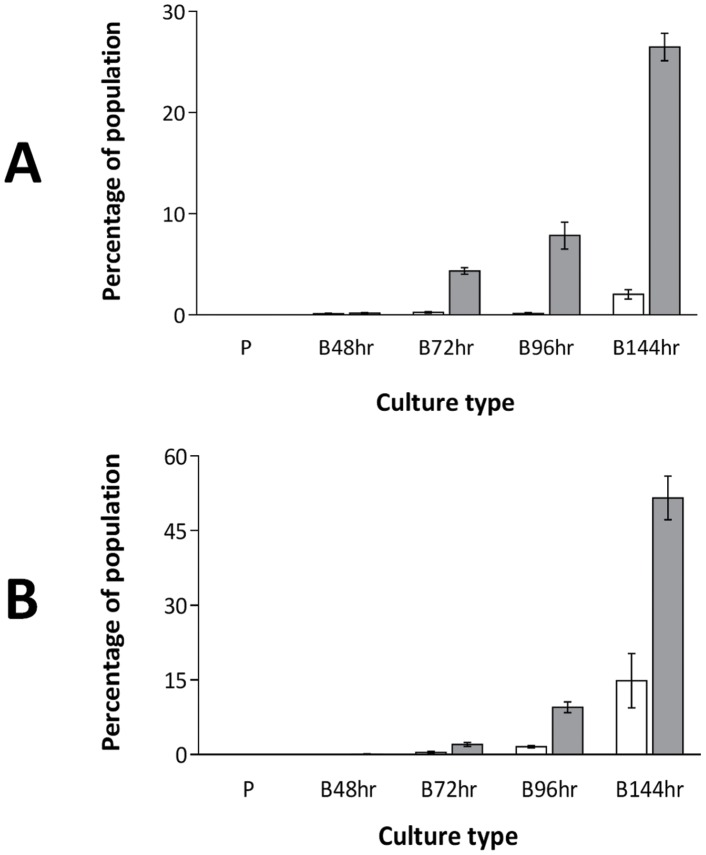
Quantification of morphological variants of *S. aureus* SH1000 arising in planktonic cultures (denoted by ‘P’) and CDS biofilms (denoted by ‘B’). (A) WVs and (B) LPVs (white and grey bars indicate the proportion of variants present in the adherent and nonadherent phase, respectively). Data are the means of three experimental replicates, and error bars indicate standard error.

Quantification of the occurrence of these variants in CDS biofilms of *S. aureus* SH1000 revealed their presence in both the adherent and nonadherent populations of the biofilm, with the majority present in the nonadherent phase (Figure 2A & 2B). After 144 hrs of biofilm growth, WVs were found to comprise 2% and 26% of the total cells of the adherent and nonadherent populations, respectively (Figure 2A). At the same time point, LPVs comprised 15% and 52% of the adherent and nonadherent populations, respectively (Figure 2B). WVs and LPVs were also detected in biofilms of *S. aureus* SH1000 grown for 96 hrs in a constant flow system, indicating that the appearance of LPVs and WVs is a general feature of staphylococcal biofilm growth, and is not uniquely associated with biofilms forming under static conditions. By contrast, WVs were not recovered from planktonic cultures after either standard (18 h) or extended (144 h) growth intervals; LPVs were not detected at 18 h planktonic growth but were present at low frequency (∼1% of the population) after 144 h growth.

Representative variants for each morphological type (WV1, WV2 and LPV1, LPV2) were selected for further characterization. Both the WV and LPV phenotypes were found to be stable upon subculture, even after multiple passages (>20), implying that the phenotypes were the result of stable genetic change(s).

### Characterisation of WVs

**Figure 3:**
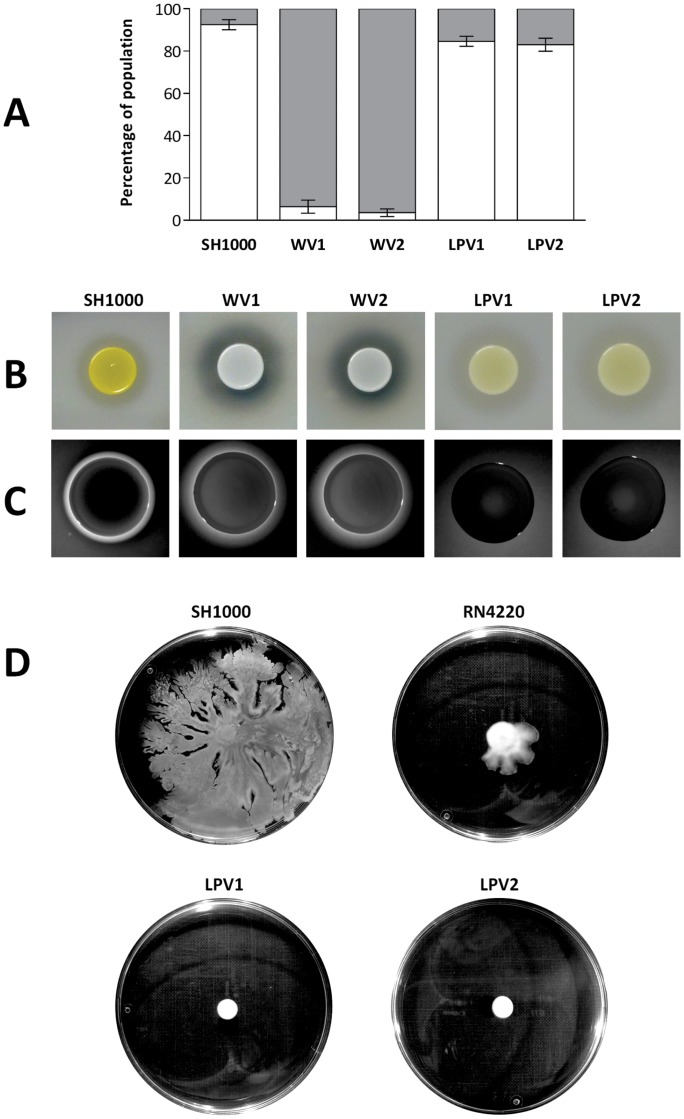
Selected phenotypic properties of *S. aureus* morphological variants identified in this study. (A) Assessment of biofilm formation in SH1000 and the variants WV1, WV2, LPV1 and LPV2 (white and grey sectors indicate proportions of adherent and nonadherent cells, respectively), (**B**) casein proteolysis of milk agar, (**C**) haemolysis of fresh blood agar, and (**D**) colony spreading. Error bars indicate standard error for three experimental replicates.

The biofilm-forming capacity of the WVs was compared with that of the parental strain (Figure 3A). WVs had lost the ability to adhere in the cellulose disk system, with only 6% and 4% of the population found in the adherent phase for WV1 and WV2, respectively (Figure 3A). Since extracellular proteases are known to inhibit biofilm formation and degrade established biofilms in *S. aureus* [23], we examined whether increased proteolysis by the WVs might play a role in their observed inability to form biofilms. In experiments assessing casein proteolysis on milk agar, WVs were found to be highly proteolytic with respect to both the parental strain and the LPVs (Figure 3B).

The nonpigmented phenotype of the WVs indicated loss of staphyloxanthin production, the biosynthesis of which is dependent upon the alternative sigma factor, SigB [24]. This sigma factor also acts to repress the synthesis of extracellular proteases and α-haemolysin [24]. Thus, lack of staphyloxanthin production, enhanced proteolysis and increased α-haemolysis on fresh blood agar compared with the parental strain (Figure 3C) were all indicative of SigB dysfunction. Accordingly, *sigB* and the gene encoding the SigB regulator (*rsbU*), were amplified by PCR from both WVs and SH1000, and subjected to DNA sequencing. Both WVs harbored alterations in *sigB* compared to the parental strain; WV1 had a 163 bp deletion that included *sigB* nucleotides 650–770, resulting in truncation of the encoded sigma factor from C_217_ onwards, whilst WV2 contained a missense mutation in *sigB* (T_724_C), encoding substitution L_242_P. Both of these mutations impact the putative DNA-binding domain of the sigma factor, which spans residues 208–247 [25].

### Characterisation of LPVs

The biofilm forming capacity of the LPVs was investigated in the CDS model (Figure 3A). LPV1 and LPV2 formed biofilms with similar proportions of adherent and nonadherent cells to the parental strain. Specifically, 85% and 83% of the total cell population was found to be adherent for LPV1 and LPV2, respectively (Figure 3A). Furthermore, no difference in proteolytic activity was observed for the LPVs compared with the parental strain (Figure 3B).

The genetic basis of the LPV phenotype was determined by subjecting LPV1 and LPV2 to whole genome sequencing. The resulting data exhibited >99.9% coverage of the *S. aureus* SH1000 genome with an average depth of 110. Both LPV1 and LPV2 harboured the same missense mutation in *agrA* (G_85_A), resulting in amino acid substitution D_29_N in the response regulator protein, AgrA. In addition, LPV2 possessed a missense mutation (G_319_A) in the gene encoding dihydropteroate synthase (SAOUHSC_00489), leading to amino acid substitution A_107_T. Both mutations were confirmed by PCR amplification and DNA sequencing. The genotypes of LPV1 and LPV2 suggested that these strains had potentially lost a functioning *agr* quorum sensing (QS) system, since substitution D_29_N alters the putative active site of AgrA [26]. To examine this, both strains were tested for two phenotypes that have been linked to *agr* dysfunction; loss of haemolytic activity [16] and failure to exhibit colony spreading [27]. Both LPVs were found to be non-haemolytic and deficient in colony spreading compared with the parental strain and the WVs (Figure 3C), indicating loss of *agr* function in these strains.

## Discussion

In this study we have identified and characterized two distinct morphological variants that are not commonly present in planktonic cultures of *S. aureus*, but which represent major subpopulations of the staphylococcal biofilm.

WVs did not produce staphyloxanthin, had lost the ability to form biofilms and displayed increased proteolysis and haemolysis; all of these phenotypes could be attributed to loss-of-function mutations detected in *sigB*. Since SigB negatively regulates the activity of the *agr* system, loss of SigB activity in WVs results in enhanced *agr* activity [23], which in turn leads to upregulation of extracellular factors such as proteases, nucleases and haemolysins, and downregulation of adhesins [28]. The loss of biofilm-forming activity in WVs is presumably the result both of reduced adhesin production (resulting in reduced adherence), and increased degradation of extracellular factors involved in formation of an extracellular matrix (e.g degradation of proteinaceous components and extracellular DNA by proteases and nucleases, respectively [29], [30], [31]).

In contrast, LPVs retained the ability to form biofilms, and were non-haemolytic and deficient in colony spreading as a consequence of loss-of-function mutation in *agrA.* LPV2 also carried a missense mutation in the dihydropteroate synthase gene; however, the absence of mutation at this locus in LPV1 indicates that it is not a requirement for the LPV phenotype. Strains with a defective *agr* quorum sensing (QS) system are more prolific biofilm formers owing to reduced production of surfactants such as phenol soluble modulins [PSMs] [32], enhanced cellular aggregation as a consequence of elevated expression of protein A [33], and reduced extracellular protease production [30]. Indeed, in wild-type strains of *S. aureus*, repression of the *agr* system is required for protein-dependent biofilm formation [30].

A previous study reported the emergence of variants with altered haemolytic properties from the staphylococcal biofilm, including both non-haemolytic and hyper-haemolytic strains [7]. These variants are apparently distinct from those that we have described here; in contrast to the LPVs, the non-haemolytic variants previously reported displayed a non-pigmented phenotype when grown on complex medium, whilst in contrast to the WVs, the hyper-haemolytic variants were phenotypically unstable [7].

The high proportion of WVs and LPVs detected in our biofilm models indicates that these variants are under strong selection, but the circumstances driving their selection are not clear. One possibility is that WVs are favoured in the early stages of staphylococcal biofilm maturation because they overproduce α-haemolysin, a protein which has been shown to mediate cell-to-cell contact and is essential for staphylococcal biofilm formation [34]. Alternatively, WVs may become selected owing to their dissemination-inducing phenotype; dissemination appears to be important for bacterial populations during biofilm growth, enabling population dispersal and survival [35], and subpopulations exhibiting an enhanced detachment phenotype have been detected in the pseudomonal biofilm [11]. WVs appear to arise earlier and in greater number than LPVs in the biofilm (Figure 3), and we propose that it is the emergence of the former that drives the appearance of the latter. The presence of a large subpopulation of cells in the biofilm exhibiting enhanced activity of the *agr* system (WVs) will allow the emergence of ‘cheater’ cells (LPVs); a subpopulation of QS negative mutants that exploits the exoproducts of QS proficient cells, and thereby gains a competitive advantage through reduced energy expenditure. An analogous form of ‘cheating’ by QS negative mutants has been reported in strains of *P. aeruginosa* [36], [37], [38].

**Figure 4:**
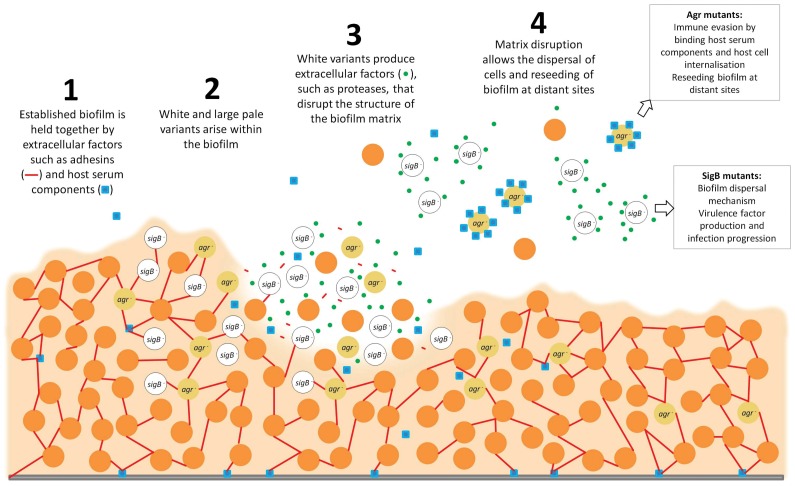
Schematic representation of the emergence and role of morphological variants in *S. aureus* biofilms during infection.

*S*. *aureus* strains with loss-of-function mutations in either *sigB* (WV) or *agr* (LPV) are known to be relevant in human infection, and both genotypes have been isolated from patients [39], [40]. The increased haemolytic and proteolytic activity in *sigB* mutants is likely to enhance virulence by increasing host cell lysis and tissue degradation. By contrast, *agr-*defective mutants show an enhanced ability to evade the host’s immune system during infection, presumably owing to a reduction in secreted virulence factors such as PSMs which strongly activate the immune response [41]. Indeed, persistent staphylococcal infections have, in some instances, been linked with strains exhibiting reduced expression of the *agr* system or defects in *agr* [42]. Our findings suggest that biofilms could potentially represent a rich source of both *sigB* and *agr* defective strains during infection, with important implications for chronic staphylococcal biofilm infections. The emergence within the biofilm of *sigB* deficient strains (WVs) could act to drive local disassembly of the biofilm, thereby facilitating release of cells from the biofilm and dissemination throughout the host. A hypervirulent subpopulation of cells released from the biofilm (WVs) may act to maximise host damage, whilst the *agr* defective cells (LPVs) emerging from the biofilm may facilitate the establishment of a persistent infection. Our concept for the emergence and release of *sigB*/*agr* mutants from the biofilm, and their contrasting roles in infection, is summarised in Figure 4. Future studies should confirm the presence of both subpopulations in biofilms formed *in vivo* during infection.
